# Microchannels Fabrication in Alumina Ceramic Using Direct Nd:YAG Laser Writing

**DOI:** 10.3390/mi9080371

**Published:** 2018-07-27

**Authors:** Muneer Khan Mohammed, Usama Umer, Ateekh Ur Rehman, Abdulrahman M. Al-Ahmari, Abdulaziz M. El-Tamimi

**Affiliations:** 1 Advanced Manufacturing Institute, King Saud University, Riyadh 11421, Saudi Arabia; muneer0649@gmail.com (M.K.M.); alahmari@ksu.edu.sa (A.M.A.-A.); 2 Industrial Engineering Department, College of Engineering, King Saud University, Riyadh 11421, Saudi Arabia; arehman@ksu.edu.sa (A.U.R.); atamimi@ksu.edu.sa (A.M.E.-T.)

**Keywords:** alumina, microchannels, Nd:YAG laser

## Abstract

Ceramic microchannels have important applications in different microscale systems like microreactors, microfluidic devices and microchemical systems. However, ceramics are considered difficult to manufacture owing to their wear and heat resistance capabilities. In this study, microchannels are developed in alumina ceramic using direct Nd:YAG laser writing. The laser beam with a characteristic pulse width of 10 µs and a beam spot diameter of 30 µm is used to make 200 µm width microchannels with different depths. The effects of laser beam intensity and pulse overlaps on dimensional accuracy and material removal rate have been investigated using different scanning patterns. It is found that beam intensity has a major influence on dimensional accuracy and material removal rate. Optimum parameter settings are found using grey relational grade analysis. It is concluded that low intensity and low to medium pulse overlap should be used for better dimensional accuracy. This study facilitates further understanding of laser material interaction for different process parameters and presents optimum laser process parameters for the fabrication of microchannel in alumina ceramic.

## 1. Introduction

The need to develop novel fabrication methods for 3D microstructures using advanced materials are increasing day by day. Many attempts have been made to develop 3D microstructures for applications in microreactors, semiconductors, microfluidic devices, microelectromechanical systems, biochips and tissue generations. In contrast to metals and polymers, ceramics are an ideal choice in high temperature and corrosive environments with excellent wear resistance properties.

However, at the same time, these properties make ceramics difficult to manufacture by conventional techniques especially for microscale applications. In order to fully utilize the potential of ceramics in harsh environments, precise and defect-free manufacturing is essential. New fabrication methods are being developed to improve micromanufacturing of ceramics in order to meet the demands of microscale features with desirable properties.

Fabrication of ceramic microparts can be divided into three broad categories i.e., replication techniques, generative processes and material removal processes. Replication techniques make the ceramic replicas by filling mold using either ceramic powder or preceramic polymers. The ceramic material should fill the mold completely, down to the micrometer details, and must be sinterable to the desired density without cracking and fragmentation. These constraints require very fine powder or slurry preparation and the manufacture of high precision micromolds. Generating processes are mainly selective laser sintering (SLS), 3D printing and laminated object manufacturing. The main advantage of these processes is that mold is not required, and the generation is carried out automatically, i.e., computer controlled. As most of the processes were developed based on polymeric materials, adaptation to ceramic materials poses significant challenges. The ceramic microparts made by generative techniques, in general, have low green density, high residual stresses due to density gradients and surface finish mainly depends on powder size.

Traditional machining processes (mechanical tooling) are the most common methods to shape ceramic microparts. However, these processes are prone to induce cracks and other defects on the machined surface and are often associated with high cost of tooling. Non-traditional methods such as Micro electric discharge machining and Micro laser beam machining are the most popular methods to fabricate microparts. Although Micro Electric discharge machining (EDM) is less expensive, it has several drawbacks in terms of tool fabrication, tool wear and surface integrity; and it is limited to machine only electrically conductive ceramics. Micro laser beam machining (LBM) on the other hand, can cut a variety of ceramic materials and optimized process design results in good dimensional control and surface properties for the microfeatures.

Microchannels are essential features in many microscale systems such as microreactors, microchemical systems and microfluidic devices. Channels with at least one dimension in the micron range i.e., between 1 to 1000 µm can be regarded as microchannels. Kandlikar and Ring [1] proposed a general scheme for the classification of different channels based on the smallest channel dimension (*D*) as shown in Table 1.

Several attempts have been made to fabricate high aspect ratio ceramic microchannels with acceptable dimensional accuracy using replication and generative techniques. In contrast, very little attention has been paid to exploring non-traditional material removal processes like Micro-LBM for the fabrication of ceramic microchannels.

X-ray lithography and lost mold techniques were applied by Wang et al. [2] to fabricate high aspect ratio microchannels in alumina ceramics. Precise ceramic parts were obtained with fine powders. However overly fine ceramic powder i.e., less than 50 nm are not recommended as they resulted in non-uniform dispersion and accumulation. A size of around 100 nm is found to be appropriate for the alumina microchannels.

LTCC (Low temperature co-fired ceramics) were utilized by Malecha and Golonka to develop microchannels. A two-step lamination process was selected using carbon black paste and cetyl alcohol as sacrificial volume materials (SVM). Both materials resulted in almost equal microchannels contraction and does not exceed 7%. Sagging control was found to be low for wide microchannels and found to be in between 25 and 75 µm. Narrow microchannels produce better results and sagging remained below 7 µm.

Pradas et al. [3] studied microchannels development using laser in photostructurable glass ceramic (Foturan) with subsequent heat treatment and chemical etching. Microchannels with round cross sections and similar apertures were fabricated on both sides of Foturan slides. They utilized pulse energies ranging between 0.3 to 3 µJ followed by etching at a maximum speed of 33 µm/min.

A hybrid method based on stereolithography and low pressure injection molding was proposed by Knitter et al. [4]. Master models were developed using CAD and stereolithography considering the expected shrinkage while sintering. They added silicon rubber to the master models to develop negative molds for the low-pressure injection of ceramic powders. The molded ceramic parts were heated up to 1700 °C at the rate of 10 °C/min to ensure complete removal of the binder material. The developed ceramic microparts with the new method show improvements in terms of both quality and productivity.

Christian and Kenis [5] showed that the gelcasting method can be effectively applied to develop submillimeter features in ceramics. They reported that replica parts can be produced with good accuracy provided that the feature size should be more than 30 times the ceramic powder size. These findings were reported for the ceramic powders with size from 0.3 to 3.0 µm.

In another work, complex 3D ceramic microparts were developed by Bertsch et al. [6] using microstereolithography. The process was based on the development of a new resin using alumina nanoparticles (80% by weight) and polymer. They ensured the fabrication of crack-free microparts with high proportions of alumina nanoparticles regardless of volume changes during sintering. Micro and nanopatterned ceramic surfaces for biomaterials were investigated by Domanski et al. [7]. The tape casting method with different slurry compositions was selected for the preparation of the silicon molds. They reported the fabrication of different microfeatures like pillars and wells with better surface integrity and dimensional control.

Kee et al. [8] developed a new technique called Pressure Laminated Integrated Structures (PLIS) to fabricate ceramic counter-flow microchannel heat exchanger. It started with mixing the ceramic powder with suitable binders. Instead of using dies and presses, the prototype designs were machined using unfired-state blocks followed by lamination and assembling in a hydraulic press and finally sintered. Scanning electron microscopy (SEM) examination showed single crystalline ceramic with no signs of joining between individual layers.

Using Micro-ultrasonic machining(micro-USM) to fabricate microchannels in ceramic materials is also the focus of many researchers. Most of the studies are related to tool wear and form accuracies. Cheema et al. [9] fabricated borosilicate glass microchannels using micro-USM by utilizing different tool materials. They analyzed the effects of tool material, abrasive particle size and step feed on form accuracy of the microchannels. It was realized that tungsten carbide tool resulted in better form accuracy than stainless steel tool. In a similar study, Kumar and Dvivedi [10] studied quantitative relationships among tool wear, form accuracy and material removal rate while developing microchannels in borosilicate glass using rotary tool micro-USM. Longitudinal and edge rounding wear were found be the major factors affecting the process. They suggested applying longitudinal wear compensation to achieve the desired depth and form accuracy. In another study, Sreehari and Sharma [11] developed silicon microchannels using micro-USM. They analyzed the effect of viscosity of different fluids and other machining parameters on surface roughness, stray cut and overcut. It was noticed that better surface finish obtained low viscosity fluids whereas overcut and stray cut were minimized using high viscosity fluids. They also reported that machining at higher feed rates minimize surface roughness, overcut and stray cut regardless of the concentration of abrasive particles.

Nd:YAG and Excimer lasers are popular choices for researchers for doing Micro laser beam machining (Micro LBM) of ceramics. This involve working with a range of pulse duration from micro to femto seconds. Most of the research works include investigating the effects of different process parameters like pulse frequency, laser fluence, track displacement, speed of the laser beam and different scanning patterns. Efforts have been made to improve the process by controlling various performance measures like residual stresses, heat effected zones, recast layers, surface roughness, dimensional accuracy, material removal rate and power requirements.

Machining of oxide ceramics with nano and femtosecond lasers using various combinations of wavelengths and pulse duration was carried out by Ihleman et al. [12]. They observed different ablation mechanisms with nano and femtosecond lasers. Multi-photon absorption was found to be a major factor with femtosecond laser ablation, whereas plasma mediation dominates in case of nanosecond lasers.

Different optimization strategies were used by researchers mostly based on development of metal models and utilization of some evolutionary algorithms [13,14,15,16]. Micro grooves in aluminum titanate were optimized by Dhupal et al. [13]. They reported that taper angle deviations can be minimized by adjusting the lamp current. Kuar et al. [14,15] performed laser microdrilling of alumina and zirconia and studied taper angles and heat effected zones. Parametric optimization was carried out based on grey relational grade and response surfaces to minimize heat effected zones and taper for the drilled holes. The proposed optimized parameters were validated by in-house experimental results. In contrast, optimization of microturning of alumina was carried out by Kibria et al. [16] to minimize surface roughness and improve dimensional accuracy. It has been noticed that laser average power, pulse frequency and scanning speeds are major parameters to reduce surface roughness and dimensional errors.

Microfeatures on zirconia ceramics using pico and nanosecond lasers were developed by Parry et al. [17]. They estimated flexural strength of ceramic parts up to 1340 MPa with good finish and crack free surfaces. Pham et al. [18] developed 3D alumina and silicon nitride microparts. Effects of lamp current, pulse width, frequency and beam travel speed on surface roughness, material removal rate and dimensional accuracy were investigated. They recommended low pulse width and beam travel speed for low surface roughness and better dimensional control. Nanosecond fiber lasers were utilized by Preusch et al. [19] to machine alumina and aluminum nitride ceramics. They reported material removal rates of 94 and 135 mm^3^/h for alumina and aluminum nitride respectively with a laser fluence of 64 J/cm^2^.

From the above literature review it can be concluded that studies related to microchannels developments, especially for structural ceramics, are rare and there is a need to further explore and assess the capabilities of micromachining processes like Micro LBM in this regard. This work demonstrates the capabilities of the Nd:YAG laser to fabricate microchannels with different aspect ratios in Alumina ceramic. The effects of laser power intensity, pulse overlap and scanning pattern on material removal rate and dimensional accuracy have been investigated and optimum parameters are reported by using grey relational grade analysis. The novel aspects of the study revealed by utilizing different scanning patterns, which is mostly overlooked by other researchers. The effects of using different scanning patterns on taper profile has been discussed. In particular, the effects of scanning patterns on ablated material removal efficiency have never been discussed and reported. In addition, depth of material removed per laser scan (*d_s_*) is also identified as an important performance measure and its relation to input variables, dimensional accuracy and material removal rate (MRR) has also been investigated. Based on the analysis optimum values of *d_s_* are reported for Alumina ceramic.

## 2. Materials and Methods

**Figure 1:**
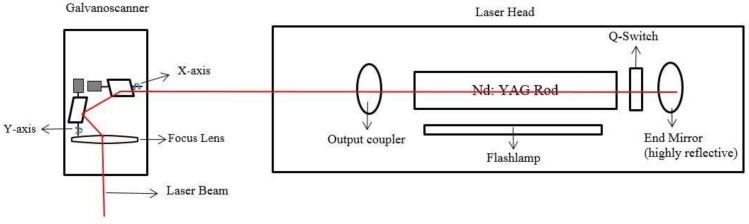
Schematic diagram of Laser.

The experiments were carried out on a Lasertec-40 machine from Deckel Maho Gildemeister, Bielefeld, Germany. The machine is equipped with a 1064 nm wavelength, Neodymium-doped Yttrium Aluminum Garnet (Nd:YAG) pulsed laser with a maximum average power of 30 W. The laser beam was used in fundamental Gaussian mode (TEM_00_) and focused on the workpiece using an optical system as shown in Figure 1. A 10 mm thick Aluminum Oxide (Al_2_O_3,_ 99.7%) was used as a workpiece material.

**Figure 2:**
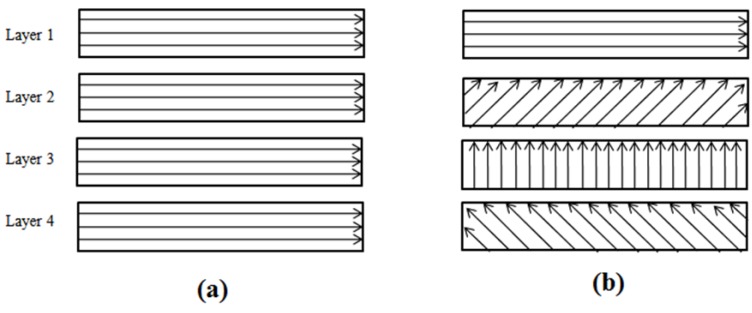
Laser beam scanning patterns (**a**) S1 (**b**) S2.

**Figure 3:**
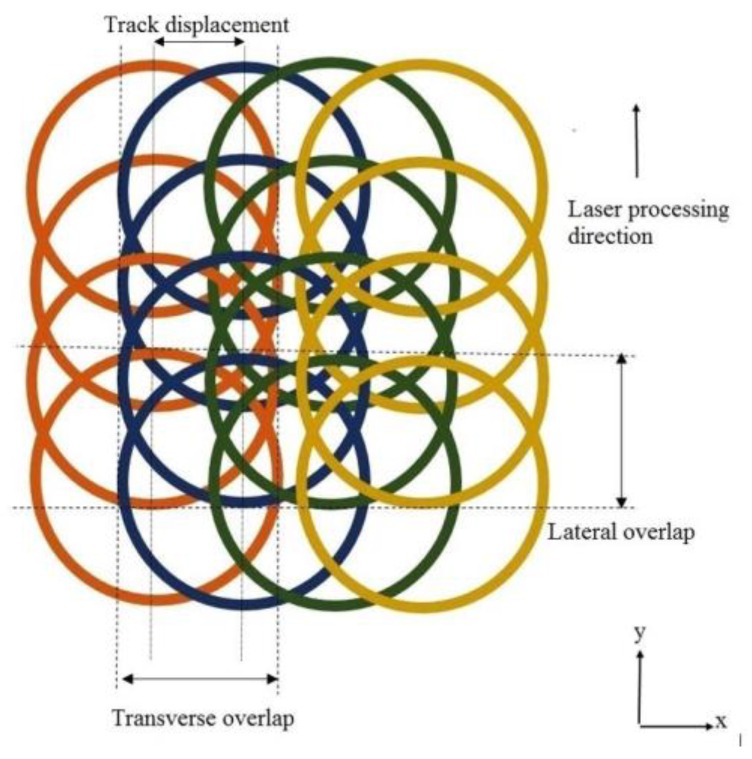
schematic diagram of pulsed laser scanning showing both the lateral and transverse overlaps for a typical 67% pulse overlap.

The microchannels were machined using two different scanning approaches, namely S1 and S2 based upon the hatch angels as shown in Figure 2. Microchannels having dimensions of 200 µm width (*w*) and 5 mm length (*l*) were planned by performing 24 laser scans (*n*) without specifying any target depth. Three parameters viz. lamp intensity, pulse overlaps and scanning patterns were selected for the design of experiment (DOE) study. Lamp intensity is related to power delivered by the laser beam on the workpiece surface. Pulse overlap represents the amount of overlap in percentage between successive pulses. Lateral overlap depends on scanning speed, pulse repetition rate and laser beam spot diameter. Transverse overlap is controlled by setting track displacement which represents the distance between two successive laser scans. In this work, both the lateral and transverse overlap were considered and kept equal by adjusting the scanning speed and track displacement as shown in Figure 3. Lateral overlap is given by:

(1) $$\;Pulse\;overlap=\;\left(1-\frac{v}{f\cdot d}\right)\times 100\;$$

where v is the scanning speed in mm/s, f is the pulse repetition rate and d is the laser spot diameter in mm. Both f and d were kept constant at 6 KHz and 30 µm respectively. Results obtained from a previous study on micro-milling of Alumina [20] were used as guidelines to select the appropriate levels for the selected factors. The pulse interaction time was set to its default machine value of 10 µs. The selected factors and their levels are shown in Table 2.

The laser machined microchannels were cut through the cross-section for scanning electron microscopy (SEM) using a Buehler Isomet^®^ precision saw. The cross-sections were analyzed using JEOL JSM-6610LV SEM (Tokyo, Japan) to measure channel top width $\left(w_{t}\right)$, bottom width $\left(w_{b}\right)$, total depth obtained (*d*) and machined area (*A*). The process performance was measured in terms of four parameters i.e., depth obtained per laser scan $\left(d_{s}\right)$, top width error ($\epsilon_{t})$, bottom width error $\left(\epsilon_{b}\right)$ and material removal rate $\left(R_{m}\right)$. They were calculated as follows:

(2) $$\;d_{s}=\frac{d}{n}\;$$

(3) $$\;\epsilon_{t}=\left(\frac{w_{t}-w}{w}\right)\times 100\;$$

(4) $$\;\epsilon_{b}=\left(\frac{w_{b}-w}{w}\right)\times 100\;$$

(5) $$\;R_{m}=\frac{Al}{t}\;$$

**Figure 4:**
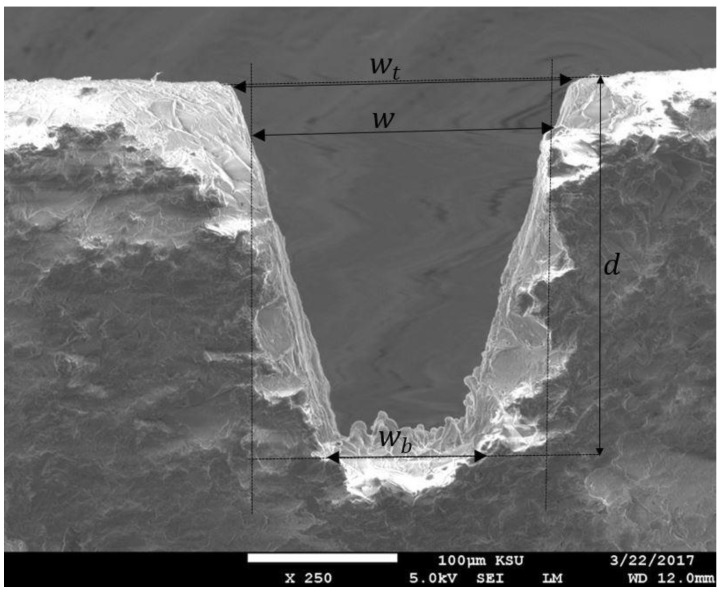
Illustration of channel’s dimensional characteristics: Top width ($w_{t}$), bottom width ($w_{b}$) and total depth (*d*).

Where *t* is the machining time for one channel and can be obtained from graphical user interface of the laser machine program. The dimensional characteristics of microchannel are shown in Figure 4. As discussed in Reference [20], the depth obtained per laser scan $\left(d_{s}\right)$ is an important performance measure in Micro LBM and significantly affects the machined workpiece surface quality. In addition, the machining time is directly proportional to the number of layers (*n*) and inversely proportional to the $d_{s}$ value. In this study the effect of $d_{s}$ on dimensional accuracy will be investigated. As stated above, no target depth was selected for the designed microchannels. In fact, once an optimum $d_{s}$ value is determined based on the selected input parameters, the total depth can be obtained by selecting the number of laser scans as per Equation (2). In this study, all performance measures were evaluated using a full-factorial design of experiments (DOE) plan which leads to 18 runs as shown in Table 2.

### Grey Relational Grade Method

A black system in grey relational analysis is one that has no information, in contrast to a white system that contains all information. A typical grey system lies somewhere in between black and white. Grey relational analysis evaluates the correlation between two sequences (experimental runs or designs) so that a comparison can be made. In situations where an experimental plan cannot be carried out as desired, grey analysis is proven to be helpful in overcoming the limitations of traditional statistical regression methods. It has the capability to handle many factors at a time and multi-objective optimization is carried out by assigning a single numerical value (grey relational grade) to each experimental run [21,22].

The first step in grey relational grade analysis is data preprocessing i.e., transforming a raw data into normalized data for comparison purposes. The procedure is called “grey relational generation”. Here the experimental results are normalized in the range between zero and one. The normalization can be carried out with different approaches depending upon the target value of the output variable. The output variable can be normalized as the-larger-the-better or the-smaller-the-better characteristic using Equations (6) and (7) respectively as [23,24]:

(6) $$\;x_{i}^{*}\left(k\right)=\frac{x_{i}^{*}\left(k\right)-\min x_{i}^{0}\left(k\right)}{\max x_{i}^{0}\left(k\right)-\min x_{i}^{0}\left(k\right)}\;$$

(7) $$\;x_{i}^{*}\left(k\right)=\frac{\max x_{i}^{0}\left(k\right)-x_{i}^{0}\left(k\right)}{\max x_{i}^{0}\left(k\right)-\min x_{i}^{0}\left(k\right)}\;$$

In case of any defined target value, the output variable should be normalized as follows:

(8) $$\;x_{i}^{*}\left(k\right)=1-\frac{\left|x_{i}^{0}\left(k\right)-x^{0}\right|}{\max x_{i}^{0}\left(k\right)-x^{0}}\;$$

where $x_{i}^{*}\left(k\right)$ is the value after normalization for the output variable *k* in the ith experimental run,$\;\min x_{i}^{0}\left(k\right)$ is the smallest value for $x_{i}^{0}\left(k\right)$, $\max x_{i}^{0}\left(k\right)$ is the largest value for $x_{i}^{0}\left(k\right)$ and $x_{i}^{0}\left(k\right)$ is the specific target value. After normalization, a grey relational coefficient is calculated for each value of output variable to express the relationship between normalized value and ideal value. The grey relational coefficient can be calculated as:

(9) $$\;\xi_{i}\left(k\right)=\frac{\Delta_{\min}+\zeta \cdot \Delta_{\max}}{\Delta_{0i}\left(k\right)+\zeta \cdot \Delta_{\max}}\;$$

where $\Delta_{0i}\left(k\right)$ is the deviation of the output variable from the reference value and can be calculated by:

(10) $$\;\Delta_{0i}\left(k\right)=∥x_{0}^{*}\left(k\right)-x_{i}^{*}\left(k\right)∥\;$$

(11) $$\;\Delta_{\max}=\underset{\forall j\in i}{\max}\;\underset{\forall k}{\max}∥x_{0}^{*}\left(k\right)-x_{j}^{*}\left(k\right)∥\;$$

(12) $$\;\Delta_{\min}=\underset{\forall j\in i}{\min}\;\underset{\forall k}{\min}∥x_{0}^{*}\left(k\right)-x_{j}^{*}\left(k\right)∥\;$$

ζ is distinguishing coefficient and a value of 0.5 is generally used. Finally, a grey relational grade ($\gamma_{i})$ for each experimental run can be calculated by taking average of grey relational coefficients of each output variable. It is given as:

(13) $$\;\gamma_{i}=\frac{1}{n}\sum \overset{n}{\underset{k=1}{\sum }}\xi_{i}\left(k\right)\;$$

The grey relational grade for each experimental run shows its relationship with respect to the reference value. The optimum parameters setting corresponds to the experimental run which has the highest grey relational grade.

## 3. Results and Discussions

All four output parameters obtained with the DOE plan are shown in Table 3. It is evident that there is considerable variation with regards to microchannels size, accuracy and material removal rate for the range of selected input parameters. For all microchannels the top width is more than the targeted value, whereas the bottom width is less than the targeted value, resulting in trapezoidal or v-shaped channels. Oversizing effects are mostly due to excess material removal across the micro-features owing to higher pulse energies and long interaction time. Undersizing at the bottom of microchannels usually occurs due to deposition of re-solidified ablated material, which further decreases the expelled efficiency of the subsequent ablated material.

**Figure 5:**
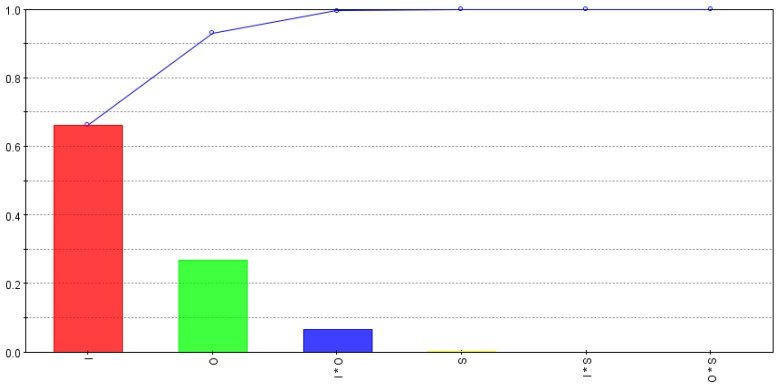
Relative strength of input variables and their interactions for depth per scan (*d_s_*).

The relative strength of input variables and their interactions for depth per scan (*d_s_*) are calculated using smoothing spline analysis of variance (ANOVA) and are shown in Figure 5. The bars show the effect of each variable and the curve shows the progressive cumulative effect up to the last variable. As shown in the figure, *d_s_* is mostly affected by intensity, overlap and combined effect of intensity and overlap. Scanning pattern and other interactions are comparatively less significant. An increase in intensity means an increase in pulse energy and an increase in overlap increases workpiece surface area subjected to multiple pulses, thus both contribute to increase in depth of material removed per laser scan.

**Figure 6:**
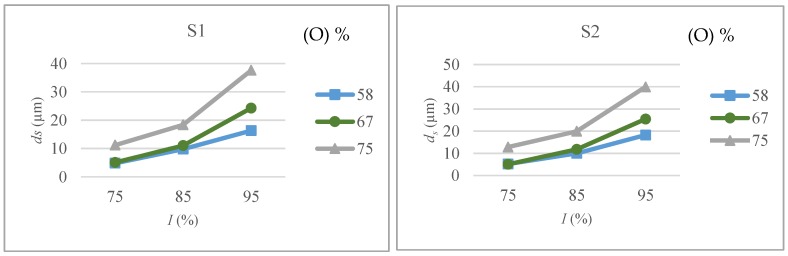
Depth per scan (*d_s_*) as a function of intensity (*I*) and overlap (*O*) for scanning pattern S1 and S2.

The variations of depth of material removed per laser scan (*d_s_*) with respect to intensity, overlap and scanning pattern are shown in Figure 6. Both scanning patterns yield similar results, except that scanning pattern S2 shows slightly higher *d_s_* values. With cross hatching pattern of S2 machining times are higher and laser scan in different directions facilitates the desired material removal. It can be inferred from the figure that change in *d_s_* with both intensity and overlap is not linear and the gradient increases sharply with increasing intensity and overlap values. Up to the 85% intensity level, change in overlap values from 58 to 67% do not show any significant increase in *d_s_* values. However, a considerable increase in *d_s_* can be observed while increasing overlap from 67 to 75%. A marked change in *d_s_* values can be observed while increasing the intensity level above 85% and any change in overlap values shows a significant effect on *d_s_* as compared to variations at low intensity regime.

**Figure 7:**
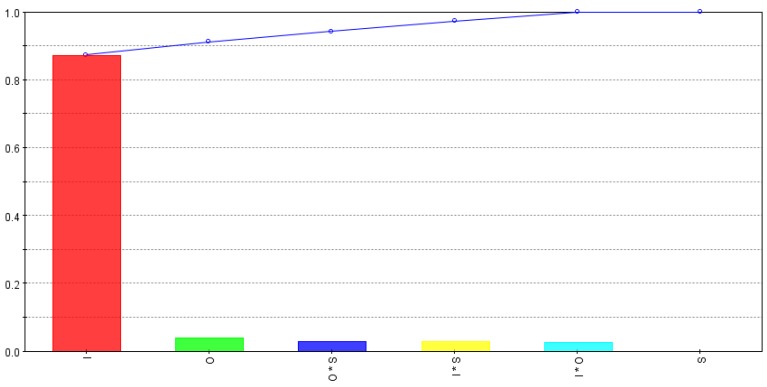
Relative strength of input variables and their interactions for top width error ($\epsilon_{t}$).

Figure 7 shows the effects of different input variables and their interactions on top width error $\left(\epsilon_{t}\right)$ of the microchannels. As shown top width error is mostly governed by intensity levels of the laser beam whereas other factors and interactions have low but almost equal impacts. In comparison to *d_s_* values, interactions effects of scanning pattern with intensity and overlap are found to be significant for variations in top width error. As the *d_s_* values are obtained by dividing the channel’s depth with the number of laser scans, the effects of pulse overlaps and scanning patterns for each laser scan may balance each other. On the other hand, the top width error $\left(\epsilon_{t}\right)$ corresponds to initial laser scans and that is why the effects of pulse overlaps and scanning patterns are higher comparatively.

**Figure 8:**
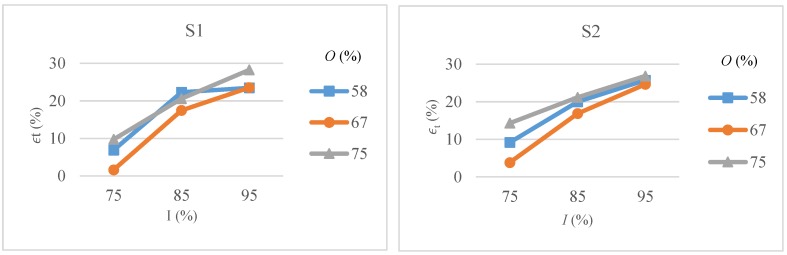
Top width error $\left(\epsilon_{t}\right)$ as a function of intensity (*I*) and overlap (*O*) for scanning pattern S1 and S2.

Variations in top width error with intensity levels and pulse overlaps for scanning pattern S1 and S2 are shown in Figure 8. It can be noted that unlike *d_s_* values, the dependence of $\epsilon_{t}$ on intensity, overlap and scanning pattern is not straightforward. For both scanning pattern and all overlap values, $\epsilon_{t}$ increases with increase in intensity levels. A medium overlap value i.e., 67% is found to give the lowest $\epsilon_{t}$ for both scanning patterns. At low overlap values, the overall irradiated surface area is larger in a given amount of time and thus more heat can be conducted to the surrounding area resulting in excess material removal. Similarly, at high overlaps the irradiated surface area is reduced but the depth of crater is increased due to multiple pulses. Excess heat concentration may also lead uncontrol material removal across the surrounding area. Maximum error is found to occur at high intensity and overlap values. It can also be noted that with the increase in intensity the effect of overlap diminishes especially for S2.

**Figure 9:**
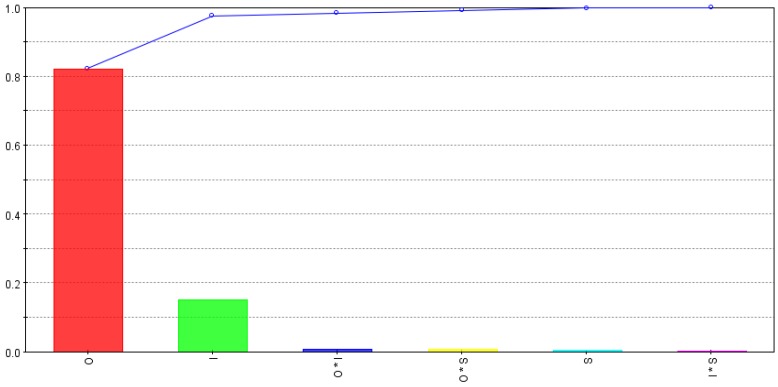
Relative strength of input variables and their interactions for bottom width error ($\epsilon_{b}$).

The relative strengths of input variable on bottom width error ($\epsilon_{b}$) of microchannels are shown in Figure 9. It is evident that pulse overlap is the dominant factor in controlling the bottom width error, followed by intensity and combined effect of intensity and overlap. Scanning pattern and its interaction with intensity and overlap are found to be comparatively less significant. Bottom width error is found to be lowest with low intensity level and low overlap values. As pulse repetition rate was kept constant, low pulse overlap means higher scanning speed of the laser beam. A combination of low pulse energy and higher scanning speed should facilitate the removal of ablated material and consequently enhances the accessibility of the laser beam for the next scan.

**Figure 10:**
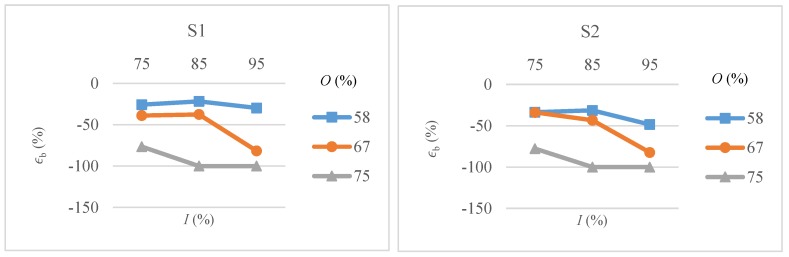
Bottom width error $\left(\epsilon_{b}\right)$ as a function of intensity (*I*) and overlap (*O*) for scanning pattern S1 and S2.

The effects of intensity overlap and scanning pattern on bottom width error of microchannels are shown in Figure 10. Results are quite similar for both scanning patterns except that S1 give a bit low error as compared to S2, particularly at low overlap values. It is clear that high pulse overlaps with intensity above 85% are giving 100% errors i.e., producing v-shaped channels. Both high intensity and high overlaps increase the thickness of ablated material and hence more material needs to be expelled in a given amount of time. As the depth of microchannel increases, the amount of resolidified ablated material increases and thus produces v-shaped channels.

**Figure 11:**
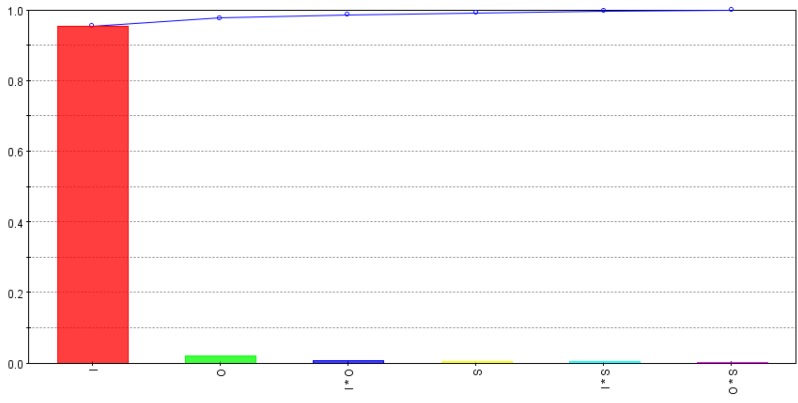
Relative strength of input variables and their interactions for material removal rate ($R_{m}$).

Figure 11 highlights the relative strengths of input variables and their interactions for material removal rate while fabricating microchannels. As shown, intensity is found to be most influential for the material removal rate followed by the pulse overlap. Scanning pattern S1 shows slightly higher material removal rates. However, interactions effects are very low and can be neglected. As shown in Figure 2, S2 requires a change in direction for the next scan and this affects the machining time as compared to S1, which comprises of parallel scan lines. As noted with *d_s_* values, the $R_{m}$ values are obtained by dividing the channel’s volume by machining time, that is why the effects of pulse overlaps and scanning patterns are negligible in comparison to intensity effects. 

**Figure 12:**
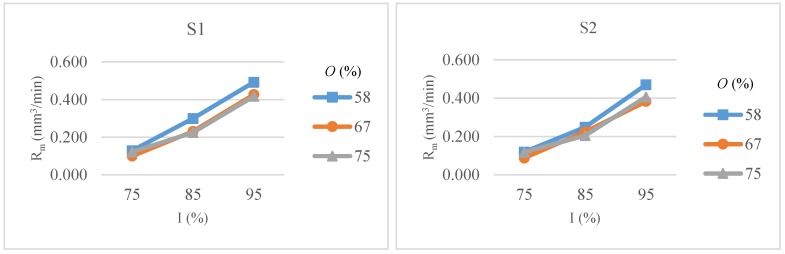
Material removal rate $\left(R_{m}\right)$ as a function of intensity (*I*) and overlap (*O*) for scanning pattern S1 and S2.

The variations in $R_{m}$ with respect to intensity, overlap and scanning pattern are shown in Figure 12. $R_{m}$ increases sharply with intensity levels for all overlaps values and scanning patterns. Results are almost similar for 67 and 75% overlap values and material removal rate is highest with high intensity and low overlap values regardless of the scanning pattern.

The reason for low dependency of $R_{m}$ on pulse overlaps might be due to the fact that although the depth of crater increases with increasing overlaps, the low scanning speed has an inverse effect on material removal rate and it decreases slightly, as shown in Figure 12.

**Figure 13:**
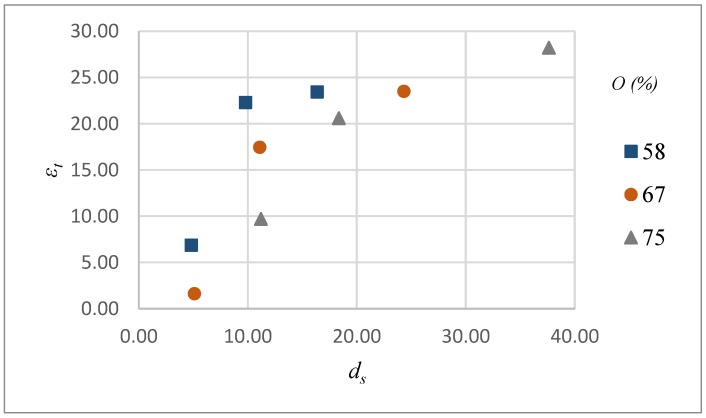
Correlation between *d_s_* and *ε_t_* at different pulse overlaps (*O*).

**Figure 14:**
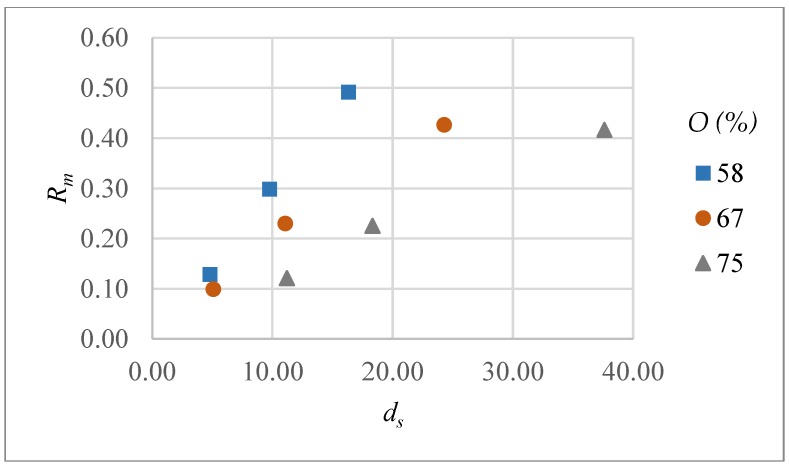
Correlation between *d_s_* and $R_{m}$ at different pulse overlaps (*O*).

Figure 13 shows a correlation between depth of material removed per laser scan (*d_s_*) and the top width error (*ε_t_*) of microchannels. For each overlap value *ε_t_* increases with *d_s_*, though the trends are different in each case. This clearly indicates that lower values of ds should be preferred to improve dimensional accuracy. However, this can lead to a low material removal rate due to an increase in number of scans required to achieve certain depth. Similarly, a correlation can be obtained between *d_s_* and the material removal rate $\left(R_{m}\right)$ as shown in Figure 14. For each pulse overlap setting, $R_{m}$ increases with *d_s_*, however the rate of change is quite different and decreases with increasing pulse overlap. Thus, *d_s_* should be considered as an important output parameter that significantly affects both dimensional accuracy and material removal rate. In other words, a decision regarding *d_s_* requires a compromise between workpiece quality and productivity.

### 3.1. Scanning Electron Microscopy (SEM) Examinations of Microchannels

**Figure 15:**
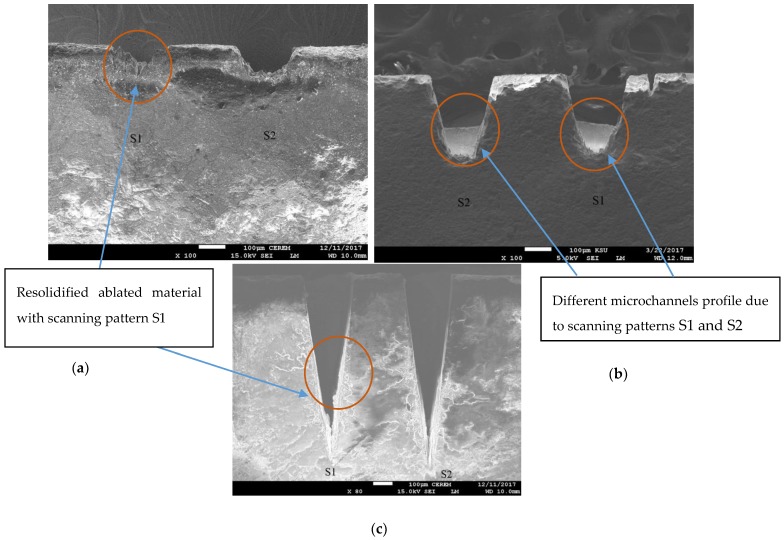
Microchannels with different depths using scanning pattern S1 and S2 (**a**) Low depth channels (**b**) Moderate depth channels (**c**) High depth channels.

Figure 15 shows low, medium and high depth channels obtained with both scanning pattern S1 and S2 using SEM photomicrographs. Low depth channels fabricated with a 75% intensity level and 58% overlap are shown in Figure 15a. It can be noted that these microchannels are associated with non-uniform material removal across the channel’s width and bottom surfaces are highly rough, especially for scanning pattern S1. The laser beam scans the material in the same direction for each layer in S1. This reduces the efficiency of the ablated material to expelled out from the microchannels resulting in deposition of resolidified ablated material as shown in Figure 15a.

Microchannels having moderate depth obtained with 85% intensity and 67% overlap are shown in Figure 15b. Although there are fewer top and bottom width errors are for S1, the bottom surface is not perfectly flat when compared with S2. In addition, the taper angle is less for S1 for the top half of the microchannel but the walls near the bottom surface can be seen with resolidified ablated material. In contrast shape of the microchannel is much better with S2, particularly across the bottom surface. This again signifies the importance of cross hatching pattern during laser scan in S2 which increases the expelled efficiency of the ablated material. Similar observations are noted with high depth channels as shown in Figure 15c. These microchannels were made with a 95% intensity level and 75% pulse overlap. As discussed previously, the bottom width error is found to be 100% with high overlaps values. High amounts of resolidified ablated material can be visualized with S1 producing rough walls in comparison to S2.

### 3.2. Grey Relational Analysis

In order to find out the optimum parameters using grey relational analysis, one has to make a decision regarding the desirable values of the output parameters. Here considering absolute values of the dimensional errors, lower values are desirable. The depth per layer target value is selected as 5.0 µm, whereas *R_m_* is considered as the-larger-the-better. The output responses were preprocessed using Equations (6)–(8) and are shown in Table 4 along with the reference values. Also, the deviation sequence for each run can be calculated by subtracting it from reference value and are shown in Table 5.

**Figure 16:**
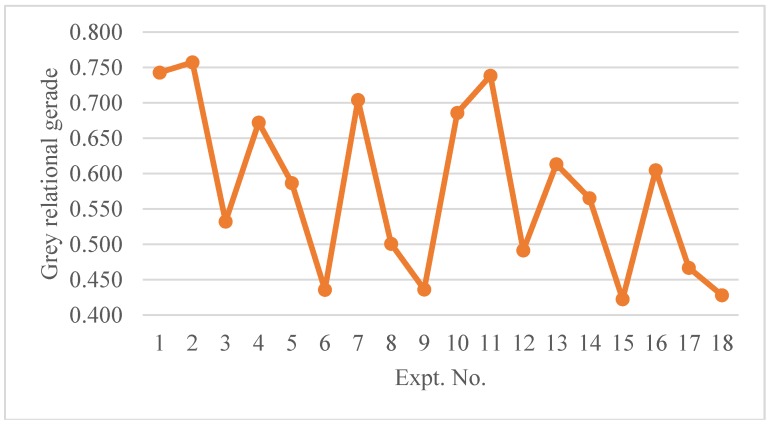
Experiments evaluation according to grey relational grade.

The grey relational grade coefficient was calculated using Equation (13). The distinguishing coefficient (ζ) was set to 0.5 while considering that all parameters have equal weighting. The grey relational coefficients and grades (γ) for each experimental run are shown in Table 6. In order to better visualize the results, the grades for each experimental run are plotted in Figure 16. It is evident that experiment no. 2 has the highest grey relational grade followed by experiment no. 1 and no. 11. These experiments are characterized by low intensity, low to moderate pulse overlap with either scanning pattern 1 or 2. On the other hand low grades are found for experiment no. 6,9,15 and no. 18. The low grades for these experiments are mainly due to 100% bottom width error i.e., the formation of v-shaped channels. All these experiments were performed with moderate to high intensities and high pulse overlap. Thus, from the experimental results and analysis it can be concluded that an intensity level of 75% and a pulse overlap of 67% gives an optimum setting for alumina microchannels. As discussed above the choice of scanning pattern significantly affects the microchannel profiles. S1 found to better especially with regard to top width error. However, the profile at the bottom are better with S2 due to a low material redeposition. A depth per scan value of 5 µm is found to be suitable and one can obtain the required depth of microchannels by selecting the number of laser scans accordingly. Also, it can be noted that *d_s_* values around 10 µm and higher are associated with high width errors and therefore should be avoided. On the other hand, very low *d_s_* i.e., around 1–2 µm are found to not be practical with alumina ceramic and to give rise to very low material removal rate. In addition, these *d_s_* values correspond to 70–72% intensity level as noted in a previous study [20] and no ablation was found at pulse repetition rate of 6 KHz and higher.

## 4. Conclusions

Microchannels formation in Alumina ceramic using Nd:YAG laser has been analyzed and a systematic approach based on grey relational grade analysis has been successfully adopted in order to find the optimum laser process parameters for the fabrication of microchannels in alumina ceramics. The main contribution of this work is therefore to give insight into the laser material interaction and provide the optimum laser process parameters, resulting in least dimensional error during the fabrication of microchannel in alumina ceramics. Overall, intensity and pulse overlap are found to be the major controlling parameters for most of the output responses. Depth of material removed per laser scan *d_s_* is found to be an important performance indicator that is strongly correlated with dimensional errors and material removal rate. Microchannels profiles are affected by the choice of scanning patterns. S1 found to give slightly less dimensional errors especially the top width error. However, the overall profile of microchannels are much better with S2. Bottom width error is found to be more dependent on pulse overlaps and increases rapidly with increase in pulse overlaps. A combination of high pulse overlap and intensity gives rise to v-shaped channels. Low intensity and low to medium pulse overlap are recommended to minimize the dimensional errors.

## Figures and Tables

**Table 1:** Channel classification scheme [1].

Conventional Channels	D>3 mm
Minichannels	3 mm≥D>200 μm
Microchannels	200 μm≥D>10 μm
Transitional Microchannels	10 μm≥D>1 μm
Transitional Nanochannels	1 μm≥D>0.1 μm
Nanochannels	0.1 μm>D

**Table 2:** Selected factors and their levels.

Factor	Symbol	Levels		
		1	2	3
Lamp Intensity (%)	*I*	75	85	95
Pulse Overlap (%)	*O*	58	67	75
Scanning Pattern	*S*	S1	S2	

**Table 3:** Experimental plan using full-factorial approach and performance measures values.

No.	S	I	O	$d_{s}\;$	$\epsilon_{t}\;$	$\epsilon_{b}\;$	$R_{m}\;$
		(%)	(%)	µm (µm)	(%) (µm)	(%)	mm^3^/min
1	1	75	58	4.812	6.888	−25.695	0.128
2	1	75	67	5.087	1.640	−38.960	0.099
3	1	75	75	11.198	9.740	−76.417	0.121
4	1	85	58	9.765	22.320	−21.694	0.299
5	1	85	67	11.078	17.459	−37.546	0.230
6	1	85	75	18.340	20.605	−100.000	0.225
7	1	95	58	16.338	23.463	−29.640	0.492
8	1	95	67	24.304	23.520	−81.521	0.426
9	1	95	75	37.614	28.233	−100.000	0.417
10	2	75	58	5.192	9.170	−33.697	0.119
11	2	75	67	5.038	3.841	−33.827	0.088
12	2	75	75	12.813	14.312	−77.680	0.118
13	2	85	58	9.957	20.029	−31.411	0.248
14	2	85	67	11.749	16.886	−43.265	0.224
15	2	85	75	19.957	21.176	−100.000	0.204
16	2	95	58	18.243	25.755	−48.557	0.470
17	2	95	67	25.449	24.650	−82.264	0.382
18	2	95	75	39.919	26.882	−100.000	0.406

**Table 4:** Normalized values of each output variable and reference value.

Expt. No.	*d_s_*	$\epsilon_{t}$	$\epsilon_{b}$	$R_{m}$
Ref. Value	1.000	1.000	1.000	1.000
1	0.995	0.803	0.949	0.099
2	0.998	1.000	0.780	0.028
3	0.823	0.695	0.301	0.082
4	0.864	0.222	1.000	0.522
5	0.826	0.405	0.798	0.352
6	0.618	0.287	0.000	0.340
7	0.675	0.179	0.899	1.000
8	0.447	0.177	0.236	0.838
9	0.066	0.000	0.000	0.814
10	0.995	0.717	0.847	0.076
11	0.999	0.917	0.845	0.000
12	0.776	0.523	0.285	0.074
13	0.858	0.309	0.876	0.396
14	0.807	0.427	0.725	0.336
15	0.572	0.265	0.000	0.287
16	0.621	0.093	0.657	0.945
17	0.414	0.135	0.227	0.729
18	0.000	0.051	0.000	0.787

**Table 5:** Deviation from the normalized values for each output variable.

Expt. No.	*d_s_*	$\epsilon_{t}$	$\epsilon_{b}$	$R_{m}$
1	0.005	0.197	0.051	0.901
2	0.002	0.000	0.220	0.972
3	0.177	0.305	0.699	0.918
4	0.136	0.778	0.000	0.478
5	0.174	0.595	0.202	0.648
6	0.382	0.713	1.000	0.660
7	0.325	0.821	0.101	0.000
8	0.553	0.823	0.764	0.162
9	0.934	1.000	1.000	0.186
10	0.005	0.283	0.153	0.924
11	0.001	0.083	0.155	1.000
12	0.224	0.477	0.715	0.926
13	0.142	0.691	0.124	0.604
14	0.193	0.573	0.275	0.664
15	0.428	0.735	1.000	0.713
16	0.379	0.907	0.343	0.055
17	0.586	0.865	0.773	0.271
18	1.000	0.949	1.000	0.213

**Table 6:** The grey relational coefficients and grade for each experimental run.

Expt. No.	*d_s_*	$\epsilon_{t}$	$\epsilon_{b}$	$R_{m}$	γ
1	0.989	0.717	0.907	0.357	0.743
2	0.995	1.000	0.694	0.340	0.757
3	0.738	0.621	0.417	0.353	0.532
4	0.786	0.391	1.000	0.511	0.672
5	0.742	0.457	0.712	0.436	0.586
6	0.567	0.412	0.333	0.431	0.436
7	0.606	0.379	0.831	1.000	0.704
8	0.475	0.378	0.396	0.755	0.501
9	0.349	0.333	0.333	0.729	0.436
10	0.989	0.638	0.765	0.351	0.686
11	0.998	0.858	0.763	0.333	0.738
12	0.691	0.512	0.412	0.351	0.491
13	0.779	0.420	0.801	0.453	0.613
14	0.721	0.466	0.645	0.430	0.565
15	0.539	0.405	0.333	0.412	0.422
16	0.569	0.355	0.593	0.902	0.605
17	0.461	0.366	0.393	0.648	0.467
18	0.333	0.345	0.333	0.701	0.428
